# Complete mitochondrial genome of yellowback seabream, *Dentex hypselosomus* and phylogenetic analysis of the family Sparidae

**DOI:** 10.1080/23802359.2019.1637298

**Published:** 2019-07-12

**Authors:** Ren-Xie Wu, Yun Zhai, Su-Fang Niu, Jing Liu, Ben-Ben Miao, Fang Liu, Chun-Xiao Ou

**Affiliations:** a College of Fisheries, Guangdong Ocean University, Zhanjiang, P.R. China;; b Laboratory of Marine Organism Taxonomy and Phylogeny, Institute of Oceanology, Chinese Academy of Sciences, Qingdao, P.R. China;; c Guangdong Leizhou Rare Marine Life National Nature Reserve, Zhanjiang, P.R. China

**Keywords:** *Dentex hypselosomus*, mitogenome, phylogeny

## Abstract

The complete mitogenome sequence of *Dentex hypselosomus* was amplified by designing 15 primer pairs. The circle genome was 16,618 bp in size and the overall base composition was 27.04% of A, 26.38% of T, 17.08% of G, and 29.50 of C, with significant anti-G bias. The complete mitogenome of *D. hypselosomus* encodes 37 canonical mitochondrial genes, two non-coding regions, an L-strand replication region (OL), and a control region (D-loop). The D-loop contained termination sequence domain (TAS), central conserved domains (CSB-F, CSB-E, CSB-D, CSB-C, and CSB-A), and conserved sequence blocks (CSB-1, CSB-2, and CSB-3). Phylogenetic analysis of nine sparid species well supported the phylogenetic position of *D. hypselosomus* and revealed the phylogenetic relationship of the family Sparidae at the level of mitochondrial genomes.

*Dentex hypselosomus* Bleeker, 1854, a member of family Sparidae, commonly called yellowback seabream, is one of the most economically marine fishes on the East Asian shelf (Yoda and Yoneda 2009). In the northwestern Pacific, *D. hypselosomus* has long been considered synonym of *Dentex tumifrons* (Temminck and Schlegel, 1843) (Iwatsuki et al. 2007). It is a demersal fish that usually inhabits mud and muddy-sand bottoms in coastal water from depths of 50 to 200 m (Carpenter 2001, p. 3000), supporting an important commercial marine fishery in the East China Sea and the northern South China Sea (Liu et al. 2016. p. 222). Previous studies mainly focused on the biological characteristics and reproductive techniques of *D. hypselosomus* or *D. tumifrons* (Lu et al. 2009; Yoda and Yoneda 2009), but little is known about the genetic background. In this regard, the complete mitogenome sequence of *D. hypselosomus* was determined in this study.

A fresh *D. hypselosomus* was obtained in March 2013 from longline operating off the Guangdong Leizhou Rare Marine Life National Nature Reserve coast (20°38′45″N, 109°43′34″E), Beibu Gulf, northern South China Sea. It was preserved in 95% ethanol and deposited in Guangdong Ocean University (No. 20130331001). Total genomic DNA was extracted from the muscle tissue using phenol-chloroform method (Sambrook and Russell 1989). The complete mitogenome of *D. hypselosomus* was amplified by designing 15 primer pairs, and the whole sequence was spliced based on the results of ABI 3730XL sequencing.

The complete mitogenome of *D. hypselosomus* had a total length of 16,618 bp (GenBank accession number: MK978157), and the overall base composition was estimated to be 27.04% of A, 26.38% of T, 17.08% of G, and 29.50of C, with a slightly higher A + T content. The complete mitogenome contained 37 canonical mitochondrial genes, 2 non-coding regions, an L-strand replication region (O_L_), and a control region (D-loop). Among the 13 protein-coding genes (PCGs), *COI* and *ND4* used start codon GTG, the rest were started with the typical ATG codon (*ND1*, *ND2*, *COII*, *ATPase8*, *ATPase6*, *COIII*, *ND3*, *ND4L*, *ND5*, *ND6*, *Cytb*). Five PCGs (*ND1*, *ATPase8*, *ND4L*, *ND5*, *ND6*) were terminated with stop codon TAA, and COI ended with AGG. On the other hand, the remaining genes (*ND2*, *ATPase6, COII*, *COII*, *ND3, ND4*, *Cytb*) had incomplete stop codon TA- or T–. Through the program tRNA-can-SE (Lowe and Eddy 1997), all the 22 tRNA genes could fold into a typical cloverleaf secondary structure except for *tRNA^Ser^* (GCT) which lacked a dihydrouridine arm. The 12S and 16S rRNA genes, located between *tRNA^Phe^* and *tRNA^Leu^* and separated by *tRNA^Val^*, were 955 bp and 1699 bp, respectively. The O_L_ was located between *tRNA^Asn^* and *tRNA^Cys^*, with 34 bp in length. The D-loop was 939 bp and located between *tRNA^Pro^* and *tRNA^Phe^*. Three domains were identified in D-loop, namely, termination sequence domain (TAS), central conserved domains (CSB-F, CSB-E, CSB-D, CSB-C, and CSB-A), and conserved sequence blocks (CSB-1, CSB-2, and CSB-3).

Phylogenetic analysis was performed in MrBayes version 3.2.7a (Huelsenbeck and Ronquist 2001) based on the complete mitogenome sequences of 9 sparid species, using *Lethrinus obsoletus* (AP009165) and *Monotaxis grandoculis* (AP009166) as outgroups. The Bayesian tree (Figure 1) showed that *D. hypselosomus* firstly clustered together with two congeneric species and formed a monophyly within the family Sparidae, and then they constituted a sister-group relationship with *Acanthopagrus*, *Pagellus*, *Sparus aurata* and *Rhabdosargus sarba*. All the clades had the high Bayesian posterior probability (96.88-100%). Therefore, the present results well supported the phylogenetic position of *D. hypselosomus* and revealed the phylogenetic relationship of the family Sparidae at the molecular level.

**Figure 1.**
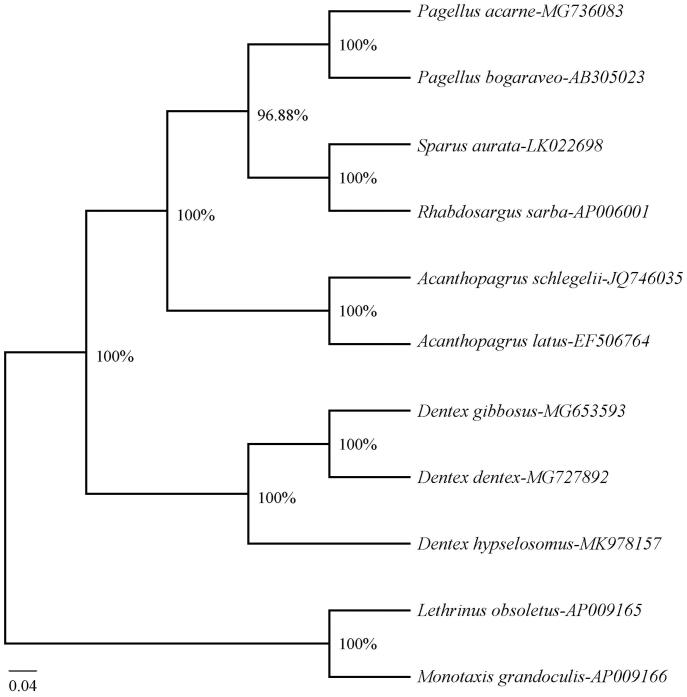
The Bayesian tree based on the complete mitogenome sequences of *Dentex hypselosomus* and other 10 species. The Bayesian posterior probability was given for each branch.
